# Impact of 11q Loss of Heterozygosity Status on the Response of High-Risk Neuroblastoma With MYCN Amplification to Neoadjuvant Chemotherapy

**DOI:** 10.3389/fped.2022.898918

**Published:** 2022-06-10

**Authors:** Xian-Ying Lu, Li-Jun Qu, Xian-Lun Duan, Wei Zuo, Kai Sai, Gang Rui, Xian-Feng Gong, Yi-bo Ding, Qun Gao

**Affiliations:** ^1^Department of General Surgery, Anhui Children's Hospital, Hefei, China; ^2^Department of Hematology and Oncology, Anhui Children's Hospital, Hefei, China; ^3^Department of Thoracic Surgery, Anhui Children's Hospital, Hefei, China; ^4^Department of Neonatal Surgery, Anhui Children's Hospital, Hefei, China

**Keywords:** neuroblastoma, MYCN, 11q, neoadjuvant chemotherapy, MIBG

## Abstract

**Purpose:**

The aim of this study was to investigate whether 11q loss of heterozygosity (LOH) aberration would impact the response of the primary tumor to neoadjuvant chemotherapy or to the degree of surgical resection in neuroblastoma (NB) patients with MYCN amplification.

**Methods:**

The clinical data of 42 NB patients with MYCN amplification who were newly diagnosed and received treatments at our hospital from 2011 to 2020 were retrospectively analyzed. According to the results of the segmental chromosome aberration analysis, the patients enrolled were assigned to an 11qLOH positive group and an 11qLOH negative group.

**Results:**

There was no significant difference in the mean number of chemotherapy courses completed before surgery between the 11qLOH positive and 11qLOH negative groups (*p* = 0.242). Each of the 42 patients had metaiodobenzylguanidine (MIBG) scans both before and after neoadjuvant chemotherapy. The percentage of patients who had a clinical MIBG change in the 11qLOH positive group was lower than the percentage in the 11qLOH negative group (27.27 vs. 66.67%, *p* = 0.030). The 11qLOH negative group seemed to have a higher rate of surgical resection (≥90%); however, the difference between the two groups was not statistically significant (*p* = 0.088). Furthermore, the 11qLOH negative group did not show significantly superior event-free survival and overall survival rates compared with the 11qLOH positive group.

**Conclusions:**

This study showed that patients with NB and MYCN amplification in combination with 11qLOH might be less likely to respond to neoadjuvant chemotherapy when compared with patients with NB and MYCN amplification without 11qLOH.

## Introduction

Neuroblastoma (NB) is the most common childhood extracranial solid tumor. Its characteristics include rapid progression, and it accounts for 8–10% of pediatric cancers (1, 2). The clinical presentation of NB can be quite heterogeneous in terms of histology and genetics, ranging from asymptomatic incidental tumors to widespread metastases with systemic manifestations (3).

Traditionally, according to age at diagnosis, stage and pathology, patients with NB are classified into low-, intermediate- and high-risk groups (1). In addition to these risk factors, the amplification of MYCN is regarded as one of the most validated and consistent prognostic markers in NB tumors (4). The amplification of MYCN is associated with the more aggressive features of NB and plays a key role in promoting the proliferation, invasion and metastasis of NB cells (5). Patients diagnosed with NB with MYCN gene amplification exhibit a poor prognosis (6, 7). In addition, targeting MYCN is a potential treatment strategy for highly vascularized NB tumors (8).

Loss of heterozygosity (LOH) at chromosome arm 11q occurs frequently in NB, with an approximate rate of 34–44% of NB samples (9, 10). Previous studies have suggested that there is an association between 11qLOH and the high-risk features of NB patients (11–13). Like MYCN amplification, 11qLOH can be used as a prognostic marker for NB (10), and it has been included as an independent risk factor in the pre-treatment risk classification of the International Neuroblastoma Risk Group (INRG) (14). However, 11qLOH and MYCN amplification are generally mutually exclusive (10, 15); MYCN amplification plus 11qLOH rarely occurs in NB samples. Interestingly, yet infrequently, some cases with MYCN amplification in combination with 11qLOH have demonstrated a dramatic decline in survival rates (14). It has been reported that some patients with MYCN amplification respond better to neoadjuvant chemotherapy (16), whereas those with 11qLOH were less likely to respond to neoadjuvant therapies (17).

It has not yet been established whether patients with MYCN amplification plus 11qLOH have a worse response to neoadjuvant chemotherapy compared with those with MYCN amplification alone. Therefore, this study aims to determine whether 11qLOH impacts the response of the primary tumor to neoadjuvant chemotherapy or the degree of surgical resection in patients with MYCN amplification.

## Methods

### Patients

This is a retrospective cohort study reviewed and approved by the Ethics Committee of our hospital. From June 2011 to April 2020, the cases of children with newly diagnosed NB who received treatments at our hospital were reviewed in this study. The inclusion criteria were as follows: (1) patients who were diagnosed with NB according to International Neuroblastoma Staging System criteria (18); (2) patients who were confirmed as having MYCN amplification by whole-genome microarray; (3) patients who received pre-operative chemotherapy; (4) patients aged 0–18 years; (5) patients who underwent segmental chromosome aberration analysis; and (6) patients with complete clinical information. Written informed consent was obtained from the children and/or their parents for publishing the data.

Patients were grouped and staged according to the INRG classification and staging system (19, 20). The clinical stage of the patients was confirmed by fluorodeoxyglucose positron emission tomography, computerized tomography and/or metaiodobenzylguanidine (MIBG) scan in addition to other methods such as bone marrow biopsy, bone scan, cranial magnetic resonance imaging and ultrasonic examination (18).

According to the results of the segmental chromosome aberration analysis, the patients enrolled were assigned to two groups: 11qLOH positive and 11qLOH negative.

### Treatment

The patients were treated in accordance with the consecutive institutional protocols for high-risk NB. All patients were treated with neoadjuvant chemotherapy and primary tumor resection. Two to six courses of chemotherapy were given before surgical resection. The neoadjuvant chemotherapy regimens included high-dose cyclophosphamide, adriamycin and vincristine (CAV) and high-dose cisplatin and etoposide (CVP) regimens, which were performed every 21 days.

### Data Collection and Evaluation

All patients were contacted for follow up through telephone calls, outpatient services, or hospitalizations until December 2021. The demographic, clinical, imaging and tumor data were extracted from the patient records. The degree of tumor resection was assessed by the surgeons as being ≥90 or <90% of the estimated pre-operative tumor volume. The metastatic disease response was assessed using an MIBG scan by comparing the post-induction Curie score with the Curie score at the time of diagnosis. A clinical MIBG change was defined as a Curie score >2 at the time of diagnosis that became ≤2 after neoadjuvant chemotherapy was administered.

The survival of the patients was also evaluated. Event-free survival (EFS) was a composite endpoint, which was defined as the time from the day of diagnosis to the day of relapse or progression of the NB, the date of death for any reason, or the last follow-up date (2). Overall survival (OS) was defined as the time from the day of diagnosis to the date of death for any reason (2).

### Statistical Analysis

SPSS software (version 22.0) was used to analyse the data in this study. Normally distributed quantitative data were described as the mean ± the standard deviation. The differences between the groups were compared with the Student's t-test. Non-normally distributed quantitative data were described as the median with range and were compared with the Mann–Whitney U test. The categorical data were described as numbers and percentages and were compared using the chi-square test or Fisher's exact test. Kaplan–Meier estimates were used in the analysis of the time-to-event variables and then compared by log–rank tests. Note that a *p* < 0.05 was considered statistically significant.

## Results

From June 2011 to April 2020, 114 patients with NB were newly diagnosed at our hospital. A total of 72 patients were excluded because of MYCN non-amplification (*n* = 64), the absence of any treatment (*n* = 5) and insufficient clinical information (*n* = 3). Finally, 42 patients with a median age of 17 months (range: 1–108 months) at the time of diagnosis were enrolled in this retrospective study. The primary tumor sites of 33 (78.57%) patients were in the retroperitoneal or adrenal regions, and nine tumor sites (21.43%) were in the mediastinum region. Thirty-six patients (85.71%) had stage IV disease, and their metastatic sites included the bone marrow (24/36), bone (22/36), distant lymph nodes (12/36), liver (4/36), lung (2/36), canalis vertebralis (3/36) and orbit (1/36). Fourteen patients (33.33%) were assigned to the 11qLOH positive group, and 28 patients (66.67%) were assigned to the 11qLOH negative group. The comparisons of the clinical characteristics of the two groups of patients are shown in Table 1.

**Table 1:** Clinical characteristics of the 42 patients with *MYCN* amplification.

**Characteristics**	**11qLOH positive group (*n* = 14)**	**11qLOH negative group (*n* = 28)**	***p*** **value**
**Gender [*****n*** **(%)]**			0.661
Male	7 (50.00)	16 (57.14)	
Female	7 (50.00)	12 (42.86)	
**Age at diagnosis [*****n*** **(%)]**			0.047
≤ 18 months	5 (35.71)	19 (67.86)	
>18 months	9 (64.29)	9 (32.14)	
**Primary site [*****n*** **(%)]**			0.232
Retroperitoneal/adrenal	9 (64.29)	24 (85.71)	
Mediastinum	5 (35.71)	4 (14.29)	
**Stage [*****n*** **(%)]**			0.640
III	3 (21.43)	3 (10.71)	
IV	11 (78.47)	25 (89.29)	
**Maximum diameter of primary tumor at diagnosis [cm, median (range)]**	15.2 (7–28.3)	10.2 (4–26.5)	0.232
**Protocol [*****n*** **(%)]**			0.508
CAV	9 (64.29)	15 (53.57)	
CVP	5 (35.71)	13 (46.43)	
**Courses completed before surgery (mean** **±SD)**	4.40 ± 1.17	3.94 ± 1.16	0.242
**Curie score at diagnosis [*****n*** **(%)]**			0.884
≤2	3 (21.43)	4 (14.29)	
>2	11 (78.57)	24 (85.71)	
**Curie score after neoadjuvant chemotherapy [*****n*** **(%)]**			0.072
≤2	6 (42.86)	20 (71.43)	
>2	8 (57.14)	8 (28.57)	
**Clinical MIBG change**^**a**^ **[*****n*** **(%)]**			0.030
No change	8 (72.73)	8 (33.33)	
Change	3 (27.27)	16 (66.67)	
**Degree of tumor resection [*****n*** **(%)]**			0.456
<90%	6 (42.86)	5 (17.86)	
≥90%	8 (57.14)	23 (82.14)	

a *Clinical MIBG change = Curie score > 2 at diagnosis to ≤2 after induction chemotherapy; No change = Curie score > 2 at diagnosis that did not become ≤2 after induction chemotherapy*.

The mean number of chemotherapy courses completed before surgery was 4.40 ± 1.17 for the 11qLOH positive group and 3.94 ± 1.16 for the 11qLOH negative group. The difference between the two groups was not significant (*p* = 0.242, Table 1). Out of the 42 patients, five (11.90%) received two courses of neoadjuvant chemotherapy, 25 (59.52%) received 3–4 courses and 12 (28.57%) received 5–6 courses.

Each of the 42 patients had MIBG scans both before and after neoadjuvant chemotherapy was administered. In the 11qLOH positive group, 11 of the 14 (78.57%) patients had a Curie score at the time of diagnosis of >2, while 3 (27.27%) patients had a Curie score ≤2 after neoadjuvant chemotherapy. In the 11qLOH negative group, 24 of the 28 (85.71%) patients had a Curie score at the time of diagnosis of >2, while 16 (66.67%) had a Curie score ≤2 after neoadjuvant chemotherapy. The percentage of patients who had a clinical MIBG change in the 11qLOH positive group was lower than in the 11qLOH negative group (27.27% vs. 66.67%, *p* = 0.030, Table 1). No patient had a Curie score of ≤2 at the time of diagnosis that increased to >2 after neoadjuvant chemotherapy.

Eight of the 14 (57.14%) patients in the 11qLOH positive group had a surgical resection greater than or equal to 90%, compared with 23 of the 28 (82.14%) patients in the 11qLOH negative group. The 11qLOH negative group seemed to have a higher rate of surgical resection greater than or equal to 90%; however, the difference between the two groups was not statistically significant (*p* = 0.088, Table 1).

**Figure 1:**
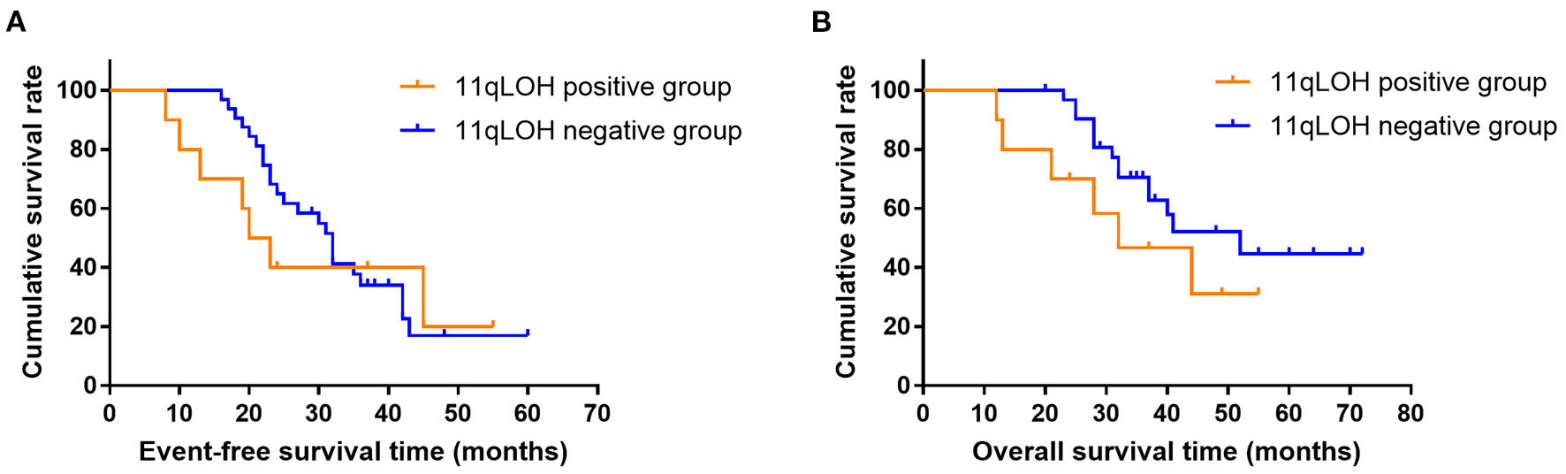
Event-free survival **(A)** and overall survival curves **(B)** for patients with MYCN amplification plus 11qLOH (*n* = 14) and MYCN amplification alone (*n* = 28).

The median EFS and OS in the 11qLOH positive group were 21.5 months and 32.0 months, respectively. The median EFS and OS in the 11qLOH negative group were 32.0 months and 52.0 months, respectively. The 11qLOH negative group did not show significantly superior EFS (Figure 1A) and OS (Figure 1B) rates when compared with the 11qLOH positive group [for EFS: Hazard ratio (HR) = 0.801, 95% confidence interval (CI): 0.325–1.973, *p* = 0.597; for OS: HR = 0.539, 95% CI: 0.175–1.655, *p* = 0.188].

## Discussion

In this study, the data from 42 patients with NB and *MYCN* amplification, treated in our hospital from 2011 to 2020, were retrospectively analyzed. The results indicated that the patients with MYCN amplification plus 11qLOH had a lower rate of clinical MIBG change after neoadjuvant chemotherapy compared with those with MYCN amplification alone. However, there was no significant statistical difference in the degree of tumor resection, EFS and OS between the two groups.

Since the 1980s, MYCN has been found to have high amplification in NB tumors, with an approximate incidence rate of 16% in all NB cases (21). MYCN amplification is an independent prognostic factor for identifying rapid tumor progression and predicting the poor prognosis of patients with NB (22). Thus, MYCN amplification is often used as a diagnostic and prognostic marker to identify high-risk NB groups (23). MYCN amplification occurs in ~40–50% of high-risk NB cases (16). For high-risk NB, the standard treatment consists of induction chemotherapy, surgical tumor resection, consolidation with single or tandem high-dose chemotherapy followed by autologous stem cell transplantation, radiotherapy and/or immunotherapy (24). Among them, induction chemotherapy plays an important role in the management of high-risk patients with NB; its goal is to obtain a maximal reduction in the tumor burden prior to the planned surgical excision (25). Previous studies have identified that MYCN amplification can be used a predictive factor for the response evaluation of induction chemotherapy or neoadjuvant chemotherapy. For instance, Campbell et al. retrospectively analyzed data from 4,672 patients with NB and found that the patients with MYCN amplification had a 25.1% complete induction response rate compared with 12.4% for patients with MYCN wild-type tumors (22). Similarly, Yanishevski et al. (16) pointed out that MYCN-amplification in high-risk NB was associated with a better response of the primary tumor to neoadjuvant chemotherapy after analyzing data from 84 high-risk patients with NB. The findings of these studies suggest that NB patients with MYCN amplification alone might have a better response to neoadjuvant chemotherapy.

Like MYCN amplification, 11qLOH has been previously identified as an adverse prognostic feature in patients with NB (23). 11qLOH is correlated with poor prognosis and the occurrence of metastatic relapses (26). Furthermore, 11q aberration has been suggested as a marker of general chemoresistance (17). 11qLOH and MYCN amplification are generally mutually exclusive (10, 15). Thus, patients with NB with both aberrations of 11qLOH and MYCN amplification are not common. In Carén's study, 37 patients with NB and MYCN amplification were enrolled, with only one patient harboring both aberrations of MYCN amplification and 11qLOH (23). In the present study, 42 patients with MYCN amplification were enrolled, 14 of which harbored an 11qLOH aberration. This study seemed to have a higher rate of MYCN amplification in combination with 11qLOH than Carén's study did. This difference may be related to the ethnic variation and the different methods used to identify the aberrations. Previous studies found that 11qLOH tumors are diagnosed at an older age (27). This study's results confirmed this finding: the 11qLOH positive group had a higher rate of patients aged >18 months at the time of diagnosis (*p* = 0.047).

Previous studies showed that the 11q status appeared to be an important determinant of response to induction chemotherapy (17). Pinto et al. (17) analyzed data from four consecutive Children's Oncology Group high-risk trials. They found that patients with 11qLOH are less likely to respond to induction therapies, while patients without 11qLOH were associated with a higher rate of end-induction response (17). Therefore, the results of these studies suggest that MYCN amplification and 11qLOH may have an opposite effect on the response to neoadjuvant chemotherapy. However, to date, whether patients with MYCN amplification plus 11qLOH had a worse response to neoadjuvant chemotherapy compared with those with MYCN amplification alone has not been determined. This study found that the percentage of patients who had a clinical MIBG change in the 11qLOH positive group was lower than that in the 11qLOH negative group after 2–6 cycles of neoadjuvant chemotherapy. This result indicates that the 11qLOH aberration may negatively affect the response of the patients with NB and MYCN amplification to neoadjuvant chemotherapy. While the patients with MYCN amplification plus 11qLOH seemed to have a higher rate of surgical resection compared with those with MYCN amplification alone, the difference between the two groups of patients was not statistically significant. Similarly, the 11qLOH negative group did not show significantly superior EFS and OS compared with the 11qLOH positive group (*p* = 0.579 and 0.188, respectively). In the future, when enough patients with the aberrations of both MYCN amplification and 11qLOH will be enrolled, statistically significant results may be obtained.

There are several limitations in this study. First, because of the retrospective nature of this study, it was not possible to collect all the necessary information. Thus, the data collected in this study were slightly incomplete. Second, the high proportion of MYCN amplification (42/114) and the high proportion of MYCN amplification combined with 11qLOH (11/42) in this study may be related to the reason that this study focused mainly on high-risk NB patients. Finally, because of the low occurrence rate of MYCN amplification in combination with 11qLOH in patients diagnosed with NB, only 14 patients with 11qLOH were included. This situation may have contributed to the results that the degree of tumor resection, EFS and OS between the two groups of patients were not statistically different. Thus, further studies with larger sample sizes and longer study periods are needed to confirm this study's results and conclusions.

## Conclusion

In conclusion, this study showed that patients diagnosed with NB, with MYCN amplification in combination with 11qLOH, might be less likely to respond to neoadjuvant chemotherapy when compared with patients with MYCN amplification alone. This result should be confirmed by future studies with larger sample sizes.

## Data Availability Statement

The original contributions presented in the study are included in the article/supplementary material, further inquiries can be directed to the corresponding author.

## Ethics Statement

The studies involving human participants were reviewed and approved by Anhui Children's Hospital. Written informed consent to participate in this study was provided by the participants' legal guardian/next of kin.

## Author Contributions

X-YL had made substantial contributions to the conception or design of the work. L-JQ and X-LD acquisition, analysis, or interpretation of data for the work. X-YL, WZ, KS, and GR have been involved in drafting the work or revising it critically for important intellectual content. X-FG and Y-bD have given final approval of the version to be published. X-YL and QG agreed to be accountable for all aspects of the work in ensuring that questions related to the accuracy of integrity of any part of the work are appropriately investigated and resolved. All authors contributed to the article and approved the submitted version.

## Funding

This work was supported by relationship between the immune function of T cell and prognosis in patients with hepatoblastoma [QG (grant number 2019xkj174)].

## Conflict of Interest

The authors declare that the research was conducted in the absence of any commercial or financial relationships that could be construed as a potential conflict of interest.

## Publisher's Note

All claims expressed in this article are solely those of the authors and do not necessarily represent those of their affiliated organizations, or those of the publisher, the editors and the reviewers. Any product that may be evaluated in this article, or claim that may be made by its manufacturer, is not guaranteed or endorsed by the publisher.
